# Mechanical deformation induces depolarization of neutrophils

**DOI:** 10.1126/sciadv.1602536

**Published:** 2017-06-14

**Authors:** Andrew E. Ekpenyong, Nicole Toepfner, Christine Fiddler, Maik Herbig, Wenhong Li, Gheorghe Cojoc, Charlotte Summers, Jochen Guck, Edwin R. Chilvers

**Affiliations:** 1 Cavendish Laboratory, Department of Physics, University of Cambridge, Cambridge CB3 0HE, UK.; 2 Biotechnology Center for Molecular and Cellular Bioengineering, Technische Universität Dresden, 01307 Dresden, Germany.; 3 Department of Physics, Creighton University, Omaha, NE 68178, USA.; 4 Klinik und Poliklinik für Kinder-und Jugendmedizin, Universitätsklinikum Carl Gustav Carus, Technische Universität Dresden, Dresden, Germany.; 5 Department of Medicine, Addenbrooke’s and Papworth Hospitals, University of Cambridge School of Clinical Medicine, Cambridge CB2 0QQ, UK.

**Keywords:** Leukocyte, priming, acute lung injury, chronic occlusive pulmonary disorder, optical stretcher, microfluidics, Neutrophil, cell mechanics, de-priming, inflammation

## Abstract

The transition of neutrophils from a resting state to a primed state is an essential requirement for their function as competent immune cells. This transition can be caused not only by chemical signals but also by mechanical perturbation. After cessation of either, these cells gradually revert to a quiescent state over 40 to 120 min. We use two biophysical tools, an optical stretcher and a novel microcirculation mimetic, to effect physiologically relevant mechanical deformations of single nonadherent human neutrophils. We establish quantitative morphological analysis and mechanical phenotyping as label-free markers of neutrophil priming. We show that continued mechanical deformation of primed cells can cause active depolarization, which occurs two orders of magnitude faster than by spontaneous depriming. This work provides a cellular-level mechanism that potentially explains recent clinical studies demonstrating the potential importance, and physiological role, of neutrophil depriming in vivo and the pathophysiological implications when this deactivation is impaired, especially in disorders such as acute lung injury.

## Teaser

In vivo–mimicking mechanical deformations quickly depolarize neutrophils—a mechanism potentially failing in acute lung injury.

## INTRODUCTION

Several biochemical agents, such as chemokines (*1*, *2*) and bacterial chemoattractants (*3*, *4*), induce the rapid transition of neutrophils from a resting state to a polarized and primed or fully activated state (*5*–*7*), leading to their transendothelial migration out of blood and recruitment to sites of infection and sterile injury in surrounding tissues (*8*). Activated neutrophils are implicated in a very broad spectrum of pathologies, especially infectious and pulmonary diseases, such as acute respiratory distress syndrome (ARDS).

Mechanical perturbations, such as shear stresses and cell stretching, can also induce activation of quiescent neutrophils, just like biochemical agents (*9*, *10*). Furthermore, shear stresses can induce the deactivation of primed and adherent neutrophils in the presence of red blood cells or superoxide dismutase (*10*–*12*). The mechanism of shear force–induced deactivation of neutrophils remains open. Because neutrophils in the circulation are repeatedly stretched, for instance, by the multiple very narrow (5 μm) capillary constrictions present in the human pulmonary microcirculation, it is unclear why not all resting neutrophils in circulation eventually become activated.

To address this question, appropriate methods to mechanically stimulate neutrophils under near-physiological conditions (suspended while circulating and without permanent attachment; many rapid, successive deformations mimicking microvascular constrictions) are required. Most current mechanical manipulation techniques (for example, micropipette aspiration, atomic force microscopy, optical tweezers, and magnetic twisting cytometry) are not appropriate (*13*) largely because they require cells to be immobilized on surfaces, which results in neutrophil activation. An attractive alternative for the noncontact study of primary neutrophils in their suspended state is a microfluidic optical stretcher (OS), where the radiation pressure from two counterpropagating laser beams is used to hold and deform cells (*14*–*17*). This technique offers the unique ability to study the dynamic response of neutrophils to chemical priming without contact and at a single-cell level, which, so far, has been unachievable with bulk measurements (*8*, *18*). The OS can also be used to apply single or continued oscillatory deformations to stationary cells, allowing a detailed study of neutrophils during priming and depriming processes.

With such an OS, we show not only that neutrophil priming can be observed directly and quantified mechanically but also that continued oscillatory mechanical deformation of primed neutrophils results in rapid depolarization. The rate at which this happens is two orders of magnitude greater than if the cells are left mechanically undisturbed. These findings are confirmed by experiments with a novel microfluidic flow device, termed microfluidic microcirculation mimetic (MMM), which leaves less time for investigating each cell in detail, but is even closer to the physiological conditions that pertain within the human pulmonary microcirculation. This work has important implications for the understanding and management of immune cell circulatory disorders, such as ARDS, which is characterized by high levels of neutrophil priming in the systemic circulation and, consequentially, high levels of neutrophil entrapment in the pulmonary microcirculation.

## RESULTS

### Chemical priming of resting neutrophils leads to characteristic molecular readouts and morphological changes

**Fig. 1:**
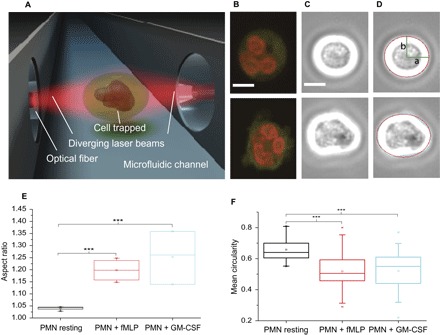
Morphological changes in activated neutrophils. (**A**) OS with two counterpropagating laser beams emanating from single-mode optical fibers, enabling contactless morphometry. (**B**) Confocal images of Syto 61 dye– and MitoTracker Orange–stained neutrophils. Resting cells remain round (top), whereas activated cells form amoeboid shapes (bottom). (**C**) Phase contrast images of cells trapped in the OS with strong white halo simplifying contour detection. (**D**) Morphometry using phase contrast images from (C). Edge detection algorithms are used to fit ellipses (red line) to the contours of the cells (cyan line), giving access to the semimajor and semiminor axes, *a* and *b*, respectively. (**E**) Plots of the aspect ratio, *a*/*b*, from the morphometry results of (D). Activated neutrophils have a significantly higher aspect ratio compared to resting neutrophils. (**F**) Mean circularity of F-actin cortex of cells stained with Alexa Fluor 488 Phalloidin. After fMLP and GM-CSF treatment, cellular circularity decreases significantly. ***P < 0.001 (significant difference). Scale bars, 5 μm. Both GM-CSF (10 ng/ml)– and fMLP (100 nM)–treated neutrophils have significantly (*P* < 0.0001) higher CD11b expression (fig. S1) and lower CD62L expression (fig. S2) compared to resting neutrophils (for details of priming, see the Supplementary Materials). In addition, intracellular ROS production was enhanced in primed neutrophils (fig. S3).

The known priming/activating effects of agents, such as *N*-formylmethionine leucyl-phenylalanine (fMLP) and granulocyte-macrophage colony-stimulating factor (GM-CSF), were accessed and quantified in the OS experiments at the single-cell level (Fig. 1A). Both GM-CSF (10 ng/ml)– and fMLP (100 nM)–treated neutrophils have significantly (*P* < 0.0001) higher CD11b expression (fig. S1) and lower CD62L expression (fig. S2) compared to resting neutrophils (for details of priming, see the Supplementary Materials). In addition, intracellular reactive oxygen species (ROS) production was enhanced in primed neutrophils (fig. S3).

There are obvious morphological consequences of priming, which can be quantified by image analysis of primed neutrophils held stationary in suspension with an OS (Fig. 1A). Resting neutrophils are spherical in shape (see Fig. 1, B to D, top), and the OS enables the detailed quantification of neutrophil size (10.39 ± 0.19 μm in diameter; *n* = 322), something that is difficult to achieve in the attached state. By comparison, human eosinophils (a small fraction of granulocytes) are slightly larger than resting human neutrophils, whereas, as expected, red blood cells are smaller (*19*). Although our method of isolating neutrophils routinely yields cells that are more than 95% pure (see Materials and Methods and the Supplementary Materials), we performed further morphological confirmation based on the unique lobulation pattern of the neutrophil nucleus. It is well established that human neutrophils have banded or segmented nuclei with three to five lobes (*19*); this unambiguously distinguishes them from the more than 200 other cell types in the human body, hence their designation as human polymorphonuclear cells (hPMNs or PMNs). We stained the nuclei using Syto 61 dye to observe the lobulation of the nuclei (Fig. 1B and figs. S1 and S2, bottom), confirming that we were exclusively studying neutrophils.

Upon priming or activation, resting neutrophils become progressively polarized and amoeboid in shape and project pseudopodia (Fig. 1, B to D, bottom). The use of an OS allowed us to study for the first time morphological priming markers in the absence of any cell contact (Fig. 1A), which could otherwise lead to artifacts (*9*, *20*). Phase contrast images of resting and activated cells, taken while the cells were trapped in the OS, can be seen in Fig. 1 (C and D). We used custom-developed edge detection algorithms to trace the edge contours (cyan line in Fig. 1F) and to fit ellipses to the images (red line in Fig. 1D), giving access to the semimajor and semiminor axes *a* and *b*, respectively. Plots of the aspect ratio, *a*/*b*, from the morphometric results (Fig. 1E) show that primed or activated neutrophils have significantly (*P* < 0.001) higher aspect ratios compared to resting neutrophils. Over the course of 30 to 120 min, the number of cells that are still primed decreases spontaneously (*4*). These morphological changes reflect the aforementioned molecular readouts (figs. S1 and S2) and can thus be seen as label-free markers of neutrophil priming. Furthermore, it is known that these morphological alterations are largely due to actin polymerization, which is, in itself, a hallmark of neutrophil activation. We therefore fixed and stained neutrophils using Alexa Fluor 488 Phalloidin (see the Supplementary Materials for details) and quantified the morphological alterations using circularity, *C*. Here, *C* = *P*^2^/4π*A*, where *P* is the perimeter and *A* is the area. *C* = 1 for a perfect circle, and *C* < 1 for amoeboid shapes. There is a statistically significant reduction (*P* < 0.001) in the mean circularity of the F-actin cortex of cells following fMLP- and GM-CSF–induced priming (Fig. 1F).

### Chemical priming causes cell mechanical changes

**Fig. 2:**
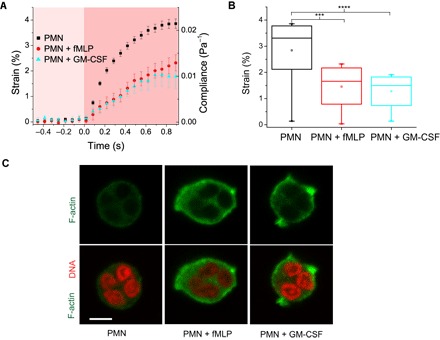
Reduced deformability of primed neutrophils in the OS. (**A**) Representative strain and compliance time course (mean ± SEM) from *n* ≥ 4 experiments for resting (*n* = 56), fMLP-treated (*n* = 24), and GM-CSF–treated (*n* =39) neutrophils. Reduction in strain and compliance is seen in GM-CSF– and fMLP-treated cells. (**B**) fMLP- and GM-CSF–treated neutrophils are significantly stiffer than resting cells [****P < 0.0001 and ***P < 0.001 (significant difference)]. (**C**) Confocal images of Alexa Fluor 488 Phalloidin–stained cells showing F-actin distribution in fixed resting and chemically activated neutrophils. Scale bar, 5 μm.

For morphological and mechanical phenotyping by the OS, cells are introduced into a microfluidic delivery system, serially trapped, and then stretched along the laser beam axis (Figs. 1A and 2A). The time-dependent strain is extracted from the video camera images, normalized by the peak stress applied and a geometric factor (*21*), and used to obtain the creep compliance (inverse of stiffness) for each cell. It should be noted that calculation of the stress applied requires knowledge of the refractive index (RI) of the cells, and we measured this (see the Supplementary Materials) using a digital holographic microscope (*22*, *23*). The average RI was 1.387 ± 0.003 with no detectable difference between the resting and activated state.

Representative strain and compliance profiles (mean ± SEM) were obtained from four independent experiments for resting, fMLP-treated, and GM-CSF–treated neutrophils (Fig. 2, A and B). Reduction in strain and compliance was seen in both GM-CSF– and fMLP-primed cells within 1 s of passive mechanical testing in the OS. The fMLP- and GM-CSF–treated neutrophils were significantly (*P* < 0.001 and *P* < 0.0001, respectively) stiffer than the resting cells. Priming or activation of neutrophils is a time-dependent phenomenon, with a progressive shape change (*4*). We have measured the mechanical properties of fMLP- and GM-CSF–treated neutrophils during the very early stages of priming when shape alterations are still amenable to accurate elliptical fits (avoiding the few spontaneously deprimed cells). Incidentally, these early stages of priming are physiologically crucial to the sequelae of activities that neutrophils undertake following exposure to the priming signal.

To ascertain whether this reduction in deformability correlates with known cytoskeletal alterations following priming, we fixed and stained neutrophils using Alexa Fluor 488 Phalloidin and imaged these cells using a confocal microscope (Zeiss LSM 700) to obtain the F-actin distribution in the cells (Fig. 2C). Even qualitatively, one can notice the higher intensity of fluorescence signals indicating increased F-actin in primed cells especially within the pseudopodia, which agrees with previous reports (*3*). Likewise, the amoeboid shape of primed cells in phase contrast images reflects the actin distribution of primed cells (Fig. 1, E and F). Thus, the OS measurements indicate that deformability is a surrogate readout of molecular and cytoskeletal modulations characterizing neutrophil priming.

### Mechanical deformation causes priming of resting neutrophils

**Fig. 3:**
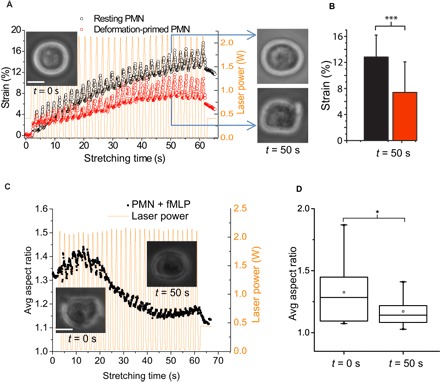
Mechanically induced priming and depolarization of neutrophils. (**A**) Multiple stretching of round, resting neutrophils in the OS using sinusoidal pulses of laser power (yellow) leads to stiffening of about 40% of the cells (red; *n* = 11) within 20 to 30 s, acquiring the primed morphology established in Fig. 1 (see inset picture at *t* = 50 s), compared to still resting cells (black; *n* = 14). Representative example of *n* = 3 experiments. (**B**) Bar graph of average strain of the two groups in (A) at *t* = 50 s. (**C**) Repeated sinusoidal pulses of laser power (*P*_max_ = 1 W per fiber; yellow) cause fMLP-primed neutrophils (inset picture of typical cell at *t* = 0 s) to depolarize, as evidenced by the retraction of pseudopods and decrease in aspect ratio (inset picture, right) (see also movies S2 and S3). The temporal evolution of the average aspect ratio of *n* = 28 cells, which is typical for *n* = 4 repeats, is shown. (**D**) Box plots of the average aspect ratio of fMLP-treated cells in (C) at *t* = 0 s and *t* = 50 s. *P < 0.05 and ***P < 0.001 (significant difference). Scale bars, 5 μm.

Mechanical perturbations can induce activation of suspended and resting neutrophils, just like soluble agents (*9*). The OS technique reproduces this effect (Fig. 3, A and B), whereby repeated stretching of unprimed cells leads to a gradual decrease in deformability and change of shape to an amoeboid “polarized” phenotype (fig. S4) in about 40% of the cells, which starts within 20 to 30 s of 0.5- to 1-s pulsatile or sinusoidal stretching (movie S1). Stretching beyond 60 s eventually engenders shape changes in an even greater proportion of cells. However, one might ask, why should soluble biological agents (for example, GM-CSF, fMLP, etc.) produce the same effect in neutrophils as do mechanical perturbations? Moreover, considering the potentially deleterious effects of inadvertent priming of neutrophils in the circulation and the presence of multiple capillary constrictions, which deform and stretch neutrophils, it is surprising that the entire circulating pool of resting neutrophils does not normally become primed by these stretching forces. We therefore investigated what might happen if the already primed neutrophils were subjected to continued, physiologically relevant deformations.

### Mechanical deformations induce depolarization of activated neutrophils

Multiple mechanical stretching with OS causes chemically (fMLP-) primed neutrophils (Fig. 3C, picture of a typical cell at *t* = 0 s) to depolarize, as evidenced by the retraction of pseudopods and subsequent decrease in their average aspect ratio (see also movie S2). With an onset at about *t* = 15 s, the neutrophils were observed to regain their round, resting shape by *t* = 50 s. About 60% of primed neutrophils (in *n* > 6 experiments) round up within 60 s of 0.5- to 1-s pulsatile stretching. Figure S5 shows the depolarization process of a typical single cell with a particularly strong activated phenotype in the same time interval. The decrease in aspect ratio toward that of resting unprimed cells is statistically significant (Fig. 3D). The same phenomenon also occurs for GM-CSF–primed neutrophils (fig. S6 and movie S3). Quite surprisingly, this deformation-induced depolarization occurred while the cells were suspended in the milieu of the priming agents (fMLP and GM-CSF) for the entire period in the OS; hence, mechanical depolarization was able to override the ongoing chemical priming stimulus. Oscillations in stress of 25% lower magnitude (fig. S7) elicit the same depolarization, albeit the return to round shape takes slightly longer. Critically, resting cells that were mechanically primed can also be depolarized by continued sinusoidal stretching (fig. S8).

Because there could be thermal effects due to the absorption of the OS laser light by water (at 1064-nm wavelength of the OS lasers and at the power of 2 × 0.9 W per fiber we used for the stretching, there should be temperature changes of about 22°C) (*24*), we undertook stretching using an OS with a 780-nm laser wavelength, where the temperature increase is only a few degrees Celsius. Under these conditions, we again found identical rounding up of primed cells (see movie S4). Thus, mechanical deformation using the OS—and not thermal effects—leads to apparent depolarization of already primed neutrophils. It should also be noted that the cells were still fully viable after mechanical depolarization and could even be chemically “reactivated/primed” (see fig. S9). We can definitively rule out that the recircularization is due to the effect of the optically induced stress. Because of the physics involved, the stress can only be tensile, that is, lead to an extension or stretching of an object along the laser axis, for example, from a sphere to a prolate spheroid, hence the name optical stretcher (*14*, *15*). If the object in the trap has already an extended, nonspherical shape, such as an activated, polarized neutrophil, it cannot be pushed into a spherical shape by the optical stresses.

Furthermore, we note that the morphological readout used in the OS is not, in itself, a formal proof of a change in other aspects of neutrophil function, including NADPH (nicotinamide adenine dinucleotide phosphate) oxidase production and degranulation responses. Unfortunately, these latter processes and other molecular marker–based metrics of depriming cannot as yet be investigated in-line at a single-cell level following optical stretching because of the low intensities of luminescence-based assays and the strong photobleaching of fluorescence-based molecular marker assays in the strong laser beams of the optical trap. However, all the morphological observations during mechanically induced depolarization closely overlap with cell changes described during neutrophil depriming (*4*, *5*). The depolarization of mechanically primed neutrophils in reaction to resolvin RvE1 also showed a similar morphological phenotype (see movie S5). The possible physiological relevance of mechanical stretching on primed circulating neutrophils can hardly be overlooked, considering that the quickest time taken for a red blood cell to transit the normal pulmonary capillary bed (which consists of 40 to 100 capillary segments) is 0.8 s, with neutrophils transiting only marginally (<3 s) slower compared to red blood cells. The capillary segments in the lung have particularly narrow constrictions (5 μm), certainly smaller than the diameter of a neutrophil (*25*). Therefore, these cells must deform at time scales close to our stretching pulses of 0.5 to 1 s. Moreover, it takes approximately 60 to 180 s for blood to circulate fully around the body. Thus, it is possible that mechanical deformation is a physiological means of effecting depriming of primed neutrophils in the circulation.

### A microcirculation mimetic confirms OS results

Circulating neutrophils in vivo deform substantially as they transit through capillary constrictions. Hence, the mechanical deformation that is experienced by circulating neutrophils in the body involves tactile squeezing by the capillary walls. However, the OS uses contactless optical forces. We therefore developed an MMM to more closely replicate the capillary constrictions that impose stretches on circulating cells in vivo.

**Fig. 4:**
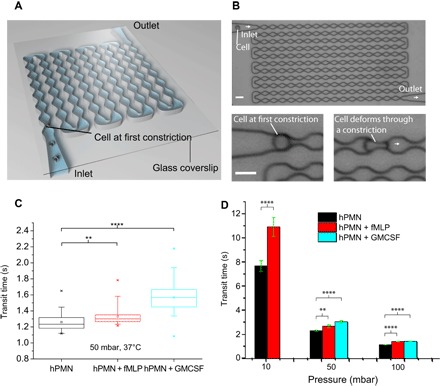
Delayed transit of activated cells in the MMM. (**A**) Schematic of the MMM, illustrating constrictions and inlet and outlet channels from and to reservoirs (not shown). (**B**) Actual micrograph of MMM showing all 187 constrictions. The minimum gaps at the constrictions are smaller than the diameter of cells, ensuring that each cell is repeatedly deformed during the advection. (**C**) Box plots of average transit times at 50-mbar driving pressure for various cell states (*n* = 50 per state), showing a statistically significant increase in transit times for all activated cells, compared to resting neutrophils. (**D**) Bar graph of average (error bar is SEM) transit times of resting, fMLP-, and GM-CSF–treated neutrophils at 37°C and pressures of 10, 50, and 100 mbar. Note that, at 10 mbar, the GM-CSF–treated cells could hardly make it through the device; hence, there are no data for this cell state at 10 mbar. **P < 0.01 and ****P < 0.0001 (significant difference). Scale bars, 15 μm.

The MMM is a polydimethylsiloxane-based device (see the Supplementary Materials for details) consisting of serpentine microchannels (Fig. 4A) that mimic the pulmonary microcirculation, with gently tapered inlet and outlet channels from and to reservoirs. This MMM differs from existing devices that model blood vessels in the lungs in that it does not involve branches or channel networks (*26*). Rather, we have maintained a serial model for two reasons. First, from the “viewpoint” of the cell, constrictions occur one after the other, branches or no branches. Second, a serial model allows us to have a very large number of constrictions (here, 187) within the field of view of the microscope objective. A constant pressure difference maintained between the inlet and the outlet, modeled on physiological pressure gradients experienced in vivo, was used to advect cells through the device, one cell at a time (see movie S5). The minimum gaps at the constrictions (5 μm) are smaller than the diameter of cells but match the size of the pulmonary capillary segments, ensuring that each cell is deformed sequentially during the advection (Fig. 4B, bottom). We used a range of physiologically relevant pressures (10, 50, and 100 mbar, that is, 10.2, 51.0, and 102 cmH_2_O, respectively) to advect the cells at 37°C and at room temperature (24°C).

Box plots and bar graphs of average transit times of resting neutrophils and fMLP-treated and GM-CSF–treated cells show that the primed cells (as would be anticipated) have significantly longer transit times than resting neutrophils, at 37°C (Fig. 4, C and D, with *P* < 0.0001) and at 24°C (fig. S10). Overall, all cells transit faster at 37°C compared to 24°C, but the relative differences in transit time between each cell state remain. Thus, there is a 20 to 40% increase in transit times between resting and primed cells at both temperatures. Figure 4D shows the transit time as a function of driving pressure for the various cell states. Note that, at 10 mbar, the GM-CSF (10 ng/ml)–treated cells could hardly transit through the device, hence the lack of data for this cell state at 10 mbar. Moreover, advecting resting neutrophils through MMM at very low pressures of 1 to 5 mbar, back and forth, led to pseudopod formation, indicating activation (movie S6). Succinctly, on the basis of transit times, there is a direct correlation between the increase in stiffness of primed cells in the OS within 1 s of stretching (Fig. 2, A and B) and the increase in transit times of primed cells in the MMM.

**Fig. 5:**
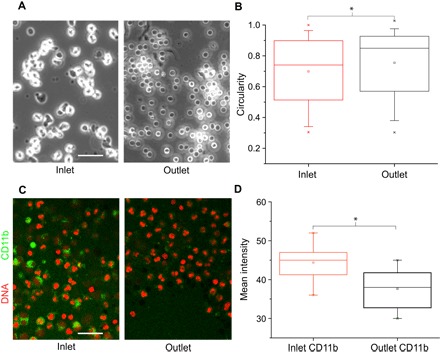
Depolarization in the MMM: shape change and molecular readouts. (**A**) Chemically primed neutrophils at the inlet showing pseudopods, round up in the outlet within 5 min following advection through constrictions. Images are representative of *n* = 5 experiments at 50-mbar driving pressure with MMM minimum gaps of 5 μm. (**B**) Box plot showing the quantification of shape changes (A), with a significant (*P* < 0.05) increase in circularity following stenotic advection for cells in the outlet, compared to cells in the inlet. (**C**) Confocal images of fMLP-treated CD11b^+^ neutrophils before (inlet) and 5 min after (outlet) stenotic advection in MMM at 50 mbar (minimum gap of 5 μm). (**D**) Box plots of CD11b intensity from (C) showing a significant reduction (*P* < 0.05) in CD11b intensity about 5 min following stenotic advection. Scale bars, 50 μm.

Furthermore, in the MMM, primed cells, following their advection (at 50 mbar) through 187 constrictions, round up in about 5 to 10 min, showing a significant increase in circularity, compared to their counterparts at the inlet reservoir (Fig. 5, A and B). This increase in circularity potentially functions as a morphological indicator of depriming since resting neutrophils are spherical. In tandem with shape change, we also found molecular evidence that the primed neutrophils, following stenotic advection in MMM, become depolarized. Hence, there was a significant decrease in the average fluorescence intensity of the CD11b signal in the postconstriction (outlet) cells compared to the preconstriction cells (inlet) (Fig. 5, C and D).

Higher CD11b expression in primed cells compared to resting cells is one of the hallmarks of priming. Thus, the decrease in CD11b expression provides further support for the view that the repeated mechanical deformation of primed neutrophils can induce their return to a resting state. As a second complimentary approach, the MMM strengthens the findings observed for single cells in the OS by also providing a link between CD11b fluorescence and depolarization, which is not possible to assess in the OS as a result of photobleaching during the laser-based trapping and manipulations.

Note that cell viability remained high after advection through the MMM in both resting and primed states (fig. S9). Cells could even be chemically reprimed after mechanical depolarization.

Chemically (GM-CSF–) primed neutrophils, which were mechanically depolarized either by optical stretching or in the MMM and then kept in the medium still containing GM-CSF, became primed again within 5 to 15 min, as evidenced by a strongly polarized cell shape. In addition, by adding fMLP, neutrophils that had been mechanically depolarized in the MMM could be activated again. These additional observations are instructive because they show that our cells remain viable after mechanical depolarization. Apoptotic or otherwise dying/dead cells would not be able to show such a physiological response. We did not observe differences of mechanically depolarized neutrophils in response to GM-CSF and fMLP. Our results therefore indicate that mechanical stimuli (deformation) can modulate the neutrophil state by priming resting cells and depolarize and/or potentially even deprime already primed or activated neutrophils in suspension. The mechanical depolarization result, which, in itself, is quite astonishing, suggests that depolarization may well happen in vivo because deformation-imposing constrictions abound in the microcirculation, especially within the lung. Accumulation of primed neutrophils is a major feature of several blood/immune system dysfunctions, such as arthrosclerosis (*27*) and chronic obstructive pulmonary disease (*28*). Can it be that these dysfunctions result, inter alia, from a failure of neutrophil depolarization?

## DISCUSSION

Neutrophils are crucial for immunity against infection, but research on them has been hampered by their experimentally difficult nature (*8*). Even slight perturbations with experimental tools alter the neutrophil state. Our use of nontactile optically generated forces in the OS enables detailed quantification of neutrophil morphology and mechanical properties as a new contact- and label-free method to assess priming. The size of resting neutrophils that we report here (10.39 ± 0.19 μm in diameter) is larger than measurements made over two decades ago (*7*, *29*) but agrees with more recent measurements, such as those of Ruban *et al*. (*30*), who report human granulocyte diameters of 9.6 ± 0.5 μm. Moreover, our measurements have been undertaken on neutrophils in suspension, which is the physiological state of these cells while in the circulation. All previous neutrophil morphometries were, as far as we are aware, carried out on cells either chemically fixed or confined to a surface or to some tactile support with the attendant obfuscation of results (*31*). The stiffening of primed compared to resting neutrophils, which we found in OS measurements, fits well with their delayed transit in the MMM. These results agree with previous reports on mechanical properties of activated neutrophils (*7*, *32*, *33*) and the delayed transit of primed neutrophils through the lungs in humans (*34*). However, when Gossett *et al*. deformed neutrophils at very high strain rates (~105 s^–1^) using deformability cytometry (DC) (*35*), they found the opposite: Primed cells were more deformable. These strain rates are known to fluidize F-actin (*36*), and DC apparently probes nuclear mechanics rather than the cytoskeleton (*35*). Otto *et al*. (*37*), using real-time DC (RT-DC), also found agonist-activated neutrophils to be more deformable than resting neutrophils. Both techniques measure cell mechanics at very short time scales: microseconds in DC and milliseconds in RT-DC. Perhaps, activated neutrophils are stiffer than resting neutrophils at longer time scales of seconds and minutes (as seen in OS and micropipette aspiration), which are physiologically more relevant to neutrophils in vivo. Functionally, increased stiffness following priming of suspended neutrophils might be useful in the tethering and rolling processes that precede the extravasation of activated neutrophils out of the vasculature into the surrounding tissues.

Multiple stretching of resting neutrophils in the OS and advection through MMM at very low pressures led to the activation of a subset of these cells, indicating that in vivo–like stretching forces can activate neutrophils. Reports of this mechanically induced priming are found in the literature (*9*). Our earlier discovery that neutrophil priming is spontaneously reversible (*4*), the ongoing search for depriming mechanisms (*38*, *39*), and the direct involvement of primed neutrophils in so many pathological states accentuate the importance of our novel finding that primed neutrophils can be depolarized by mechanical stimuli. From a broader biophysical perspective, mechanical deformation is a potent stimulus for cellular processes, such as growth, differentiation, migration, remodeling, and gene expression (*40*). We report here for the first time that whole-cell deformation depolarizes preprimed neutrophils. This is distinct from previous reports of depriming by fluid shear stress, which was insufficient to cause passive deformation of cells, acted via surface receptors only, and required the presence of erythrocytes or superoxide anion scavengers (*10*). Our single-cell investigations also provide unprecedented real-time insight into the kinetics of mechanical depolarization, which occurs within 60 s, much faster than the 30 to 120 min of spontaneous depriming stimulus (*4*), and which was inaccessible to previous bulk investigations of mechanical depriming using shear rheometers (*10*). The pathophysiological consequences of our findings seem to be emerging contemporaneously.

There is now evidence based on recent experimental medicine studies that the healthy human lung can retain or trap primed neutrophils within the pulmonary microcirculation, deprime them, and then release them into the systemic circulation (*34*). In our recent parallel clinical studies, we showed that this physiological depriming mechanism fails in patients with ARDS, resulting in increased numbers of primed neutrophils within the systemic circulation (*34*). However, how is this depriming normally effected? Because priming can be induced by chemical stimuli [for example, fMLP, GM-CSF, platelet-activating factor (PAF), and tissue plasminogen activator], finding chemical agents that cause depriming should be expected. Anti-inflammatory drugs seem to fit this model. A recent prospective study of the anti-inflammatory effects of certain drugs went as far as using loss of cell deformability, morphological changes, and increase in neutrophil elastase as measures of neutrophil priming due to incubation with the proinflammatory cytokine interleukin-8 (*41*). Change in deformability, investigated with a cell transit analyzer, was one of the three markers of priming used in the study. Notably, the drugs milrinone, piclamilast, urinastatin, ketamine, protein C concentrate, and FK 409 also have depriming or deactivating effects on neutrophils. The deactivating effects consisted of increased deformability, reduced pseudopod formation, and impaired release of neutrophil elastase (*41*). Perhaps, these drugs may also deprime neutrophils and thereby solve the problem of stiffening and retention in capillaries of patients with circulatory and inflammatory disorders. Another study reported on resolvins to stimulate rapid shape changes and stop neutrophil chemotaxis within similar time frames, as we observed for mechanically induced depolarization (*42*).

However, besides depriming reagents, how does the healthy body deprime neutrophils? Because chemical stimuli prime and seemingly deprime neutrophils, our results extend the trend with mechanical stimuli also priming and depolarizing/depriming neutrophils. Circulating neutrophils have to repeatedly squeeze through microvasculature constrictions smaller than their diameter while being advected through the pulmonary capillary bed at high speed (*25*). Although it may reflect an in vitro artifact and would need to be substantiated in vivo, our observed in vitro depolarization by deformation suggests such an in vivo mode of depolarization. Succinctly, the physiological and pathological implications of our discovery—that in vivo–mimicking deformations can depolarize activated and suspended neutrophils—may explain why circulating neutrophils do not all eventually get activated. Our finding presents neutrophil mechanotransduction as a biophysical challenge with clinical promise. Neutrophils are notoriously difficult to work with, which could be one reason why less is known about potential mechanosensitive mechanisms than for many other cell types. The main body of work on mechanical depriming of neutrophils by fluid shear stress has been contributed by the group of Schmid-Schönbein (*10*–*12*, *43*, *44*). The physical stimulus seems to be transduced by heterotrimeric guanine nucleotide–binding protein–coupled receptor down-regulation with concomitant deactivation of Rac and depolymerization of F-actin, which, together with proteolytic cleavage of β_2_ integrin, facilitates membrane detachment and lamellipodia retraction. In other cell types, Rac controls the cooperative relationship between myosin II and the actin cross-linker cortexillin I, where both proteins are essential for cellular mechanosensory responses (*45*). Rho and CDC42 do not seem to be involved (*46*). The sensitivity to fluid shear stress can also be modulated by the amount or organization of cholesterol, which changes the membrane’s physical properties (*47*). Recently, other membrane receptors, such as the formyl peptide receptor, have also been implicated in the depriming mechanism (*48*), whereas the activation with PAF can also be increased by fluid shear stress (*49*). However, the situation that pertains to the phenomena we report appears substantially different for two main reasons: (i) The fluid shear stresses used to elicit the mechanisms reported and discussed previously are “between about 1 and 10 dyn/cm^2^, a range that is below the level to achieve a significant passive, viscoelastic response” (*43*). Stresses even in the OS are between 1 and 10 Pa (10 to 100 dyne/cm^2^), an order of magnitude larger and even greater in the MMM. Thus, in our case, the entire cell is being deformed, and with it the plasma membrane, the actin cortex, the microtubule cytoskeleton, and also the nucleus. Any of these structures, which bear tensile or compressive stresses during deformation, could be involved in mechanotransduction in principle so that it is possible that mechanisms other than those described for the sensing of fluid shear stress might contribute (*50*). (ii) Cells in our case are fully suspended, with no chance to adhere to anything. This rules out any mechanism involving integrin-mediated mechanosensing involving focal adhesion complexes—likely the most important and best-studied mechanism of mechanosensing in adherent cells (*51*–*53*). The molecular mechanism behind the whole-cell deformation–induced depolarization (*19*) is now an open question, as is whether the observed depolarization actually represents depriming.

In summary, we have used several biophysical tools specifically designed to study neutrophil priming and depolarization by deformations mimicking the passage through the pulmonary microcapillary bed and have obtained results that provide novel insights into neutrophil function, with putative pathophysiological consequences. Optical stretching has afforded us a contact-free assessment of neutrophil mechanics in suspension, mimicking the circulatory environment of neutrophils. We have shown that multiple stretching of primed neutrophils in suspension leads to their rapid depolarization if not even to depriming. Because the MMM, from a pressure and size perspective, models the pulmonary capillary bed, the mechanical depolarization we have uncovered presents itself as a cellular-level mechanism for recent clinical inference that the pulmonary vasculature operates as a site for physiological depriming, with obvious pathological implications when such a depriming mechanism is impaired.

## MATERIALS AND METHODS

### Study design

This study tested the response of resting and activated neutrophils from healthy adults to in vivo–like mechanical stimuli, imposed during controlled laboratory experiments. Institutional review board guidelines of the University of Cambridge (UK) and the Technische Universität Dresden (Germany) were followed.

### Cell preparation

Primary human neutrophils were isolated from peripheral blood taken from more than 100 healthy donors at the Department of Medicine, Addenbrooke’s Hospital, University of Cambridge and the Universitätsklinikum, Technische Universität Dresden, using a method that yields basally unprimed (resting) neutrophils (*54*). Donors with regular drug intake or suspicion of infection were excluded. The purity of neutrophils obtained was routinely about 95%. Details of the preparation protocol are in the Supplementary Materials.

### Optical stretching and determination of creep compliance

The principle, setup, and handling of the OS have been described previously (*14*, *16*). The forces that trap and deform the cell outwardly along the surface arise from the change in the RI at the cell medium interface and the conservation of momentum. The axial strain during optical stretching is given by

$$\varepsilon (t)=\frac{a(t)-a_{0}}{a_{0}}$$ (1)

where *a*_0_ is the semimajor axis of the nonstretched cell and *a*(*t*) is the time-varying semimajor axis measured (Fig. 1F). The optical stress on the cells was computed using an electromagnetic wave model (*55*), which requires knowledge of the cells’ average RI. We measured the RI of resting neutrophils (see the Supplementary Materials) using a digital holographic microscope (DHM) (*22*, *23*). The resting neutrophils were immobilized using poly-d-lysine–coated slides. Because surfaces can easily activate neutrophils, we checked the DHM results by simultaneously trapping suspended resting neutrophils in the OS and measuring their RI using a quantitative phase camera (Phasics). There was no statistically significant difference in the average RI measured using DHM or the Phasics camera. Thus, the mechanical properties reported here are decoupled from the dielectric characteristics of cells.

The strain was normalized by the peak value of the calculated optical stress, σ_0_, and a geometric factor, *F*_g_, to give the creep compliance

$$J(t)=\frac{\varepsilon (t)}{\sigma_{0}F_{g}}$$ (2)

The geometric factor was calculated as described elsewhere (*21*) to account for cell shape and stress distribution.

### Fluorescence staining and confocal microscopy

Alexa Fluor 488 Phalloidin was used to treat cells fixed with 4% formaldehyde and permeabilized with 0.1% Triton X-100. For immunofluorescence, fluorescein isothiocyanate–conjugated monoclonal CD11b antibody and Pacific Blue–conjugated monoclonal CD62L were used on live cells. Nuclear staining to verify lobulation was undertaken using Syto 61 dye, whereas MitoTracker Orange was used to identify cytoplasmic regions. A confocal microscope, LSM 700 (Zeiss), was used for imaging and fluorimetry, with all settings (gain, percentage transmission, etc.) kept constant for all samples of a given experiment for valid comparisons. Further details are given in the Supplementary Materials.

### Statistical analysis

For each cell treatment condition, the number of cells per OS experiment was usually *n* > 30. Strain and creep compliance data are presented as mean ± SEM. Representative compliance data were chosen from a total number of *n* independent experiments, as indicated in the figures and figure legends. Overall, the number of individual cells analyzed totaled about 4000 for OS and about 2000 for MMM. For each cell state or treatment condition, the number of cells per advection experiment (MMM) was *n* > 40. Advection times are representative of three independent experiments and are presented as mean ± SEM. Statistical comparisons for all experiments were performed using one-way analysis of variance (ANOVA) in Origin (OriginLab Corporation) to account for multiple groups. Within ANOVA, significant differences were reported only where at least three different mean comparison tests (Tukey, Bonferroni, and Dunn-Šidák) simultaneously showed these differences. All box plots include whiskers at the 5 to 95% range, horizontal box lines at the 25 to 75% range, the median line, and the mean (inset box).

## Supplementary Material

http://advances.sciencemag.org/cgi/content/full/3/6/e1602536/DC1

## SUPPLEMENTARY MATERIALS

Supplementary material for this article is available at http://advances.sciencemag.org/cgi/content/full/3/6/e1602536/DC1 Supplementary Materials and Methods fig. S1. Chemical priming of resting neutrophils leads to increase in CD11b expression. fig. S2. Chemical priming of resting neutrophils leads to decrease in CD62L expression. fig. S3. Higher ROS production in activated neutrophils compared to resting neutrophils. fig. S4. Average aspect ratio during mechanical priming of neutrophils. fig. S5. Mechanically induced depolarization of a chemically primed neutrophil. fig. S6. Mechanically induced depolarization of GM-CSF–primed neutrophils. fig. S7. Mechanically induced depolarization of a chemically primed neutrophil at a lower laser power. fig. S8. Mechanically induced priming and depolarization of resting neutrophils. fig. S9. Viability tests using trypan blue and annexin V/propidium iodide. fig. S10. Delayed transit of activated cells in MMM at a lower temperature of 24°C. movie S1. Mechanical deformation causes priming of resting neutrophils. movie S2. Mechanically induced depolarization of PMN (fMLP-treated). movie S3. Mechanically induced depolarization of PMN (GM-CSF–treated). movie S4. Mechanically induced depolarization of PMN in OS without thermal effects. movie S5. RvE1-induced recircularization of mechanically polarized PMN. movie S6. Confirmation of OS results with MMM. movie S7. Activation of some resting cells in MMM constrictions. Other materials S1 to S3. Consent forms and questionnaire for blood donors. References (*56*–*59*)
