# Supplementary material for: Mechanical deformation induces depolarization of neutrophils
Source: Sci Adv. 2017 Jun 14;3(6):e1602536. doi: 10.1126/sciadv.1602536 (PMC5470826; doi:10.1126/sciadv.1602536)
Supplement: http://advances.sciencemag.org/cgi/content/full/3/6/e1602536/DC1 [file supp_3_6_e1602536__index.html]

Science Advances | Science Advances

## Supplementary Materials

**This PDF file includes:**

- Supplementary Materials and Methods
- fig. S1. Chemical priming of resting neutrophils leads to increase in CD11b expression.
- fig. S2. Chemical priming of resting neutrophils leads to decrease in CD62L expression.
- fig. S3. Higher ROS production in activated neutrophils compared to resting neutrophils.
- fig. S4. Average aspect ratio during mechanical priming of neutrophils.
- fig. S5. Mechanically induced depolarization of a chemically primed neutrophil.
- fig. S6. Mechanically induced depolarization of GM-CSF–primed neutrophils.
- fig. S7. Mechanically induced depolarization of a chemically primed neutrophil at a lower laser power.
- fig. S8. Mechanically induced priming and depolarization of resting neutrophils.
- fig. S9. Viability tests using trypan blue and annexin V/propidium iodide.
- fig. S10. Delayed transit of activated cells in MMM at a lower temperature of 24°C.
- Legends for movies S1 to S7
- Other materials S1 to S3. Consent forms and questionnaire for blood donors.
- References (*56–59*)

Download PDF

**Other Supplementary Material for this manuscript includes the following:**

- movie S1 (.wmv format). Mechanical deformation causes priming of resting neutrophils.
- movie S2 (.wmv format). Mechanically induced depolarization of PMN (fMLP-treated).
- movie S3 (.wmv format). Mechanically induced depolarization of PMN (GM-CSF–treated).
- movie S4 (.wmv format). Mechanically induced depolarization of PMN in OS without thermal effects.
- movie S5 (.avi format). RvE1-induced recircularization of mechanically polarized PMN.
- movie S6 (.wmv format). Confirmation of OS results with MMM.
- movie S7 (.wmv format). Activation of some resting cells in MMM constrictions.

Download Movies S1 to S7

**Files in this Data Supplement:**

- Adobe PDF - 1602536\_SM.pdf
