# Supplementary material for: Mechanical deformation induces depolarization of neutrophils
Source: Sci Adv. 2017 Jun 14;3(6):e1602536. doi: 10.1126/sciadv.1602536 (PMC5470826; doi:10.1126/sciadv.1602536)
Supplement: http://advances.sciencemag.org/cgi/content/full/3/6/e1602536/DC1 [file 1602536_SM.pdf]

advances.sciencemag.org/cgi/content/full/3/6/e1602536/DC1

Supplementary Materials for

Mechanical deformation induces depolarization of neutrophils

Andrew E. Ekpenyong, Nicole Toepfner, Christine Fiddler, Maik Herbig, Wenhong Li, Gheorghe Cojoc, Charlotte Summers, Jochen Guck, Edwin R. Chilvers

Published 14 June 2017, *Sci. Adv.* **3**, e1602536 (2017)
DOI: 10.1126/sciadv.1602536

The PDF file includes:

- Supplementary Materials and Methods
- fig. S1. Chemical priming of resting neutrophils leads to increase in CD11b expression.
- fig. S2. Chemical priming of resting neutrophils leads to decrease in CD62L expression.
- fig. S3. Higher ROS production in activated neutrophils compared to resting neutrophils.
- fig. S4. Average aspect ratio during mechanical priming of neutrophils.
- fig. S5. Mechanically induced depolarization of a chemically primed neutrophil.
- fig. S6. Mechanically induced depolarization of GM-CSF–primed neutrophils.
- fig. S7. Mechanically induced depolarization of a chemically primed neutrophil at a lower laser power.
- fig. S8. Mechanically induced priming and depolarization of resting neutrophils.
- fig. S9. Viability tests using trypan blue and annexin V/propidium iodide.
- fig. S10. Delayed transit of activated cells in MMM at a lower temperature of 24°C.
- Legends for movies S1 to S7
- Other materials S1 to S3. Consent forms and questionnaire for blood donors.
- References (*56*–*59*)

Other Supplementary Material for this manuscript includes the following:
(available at advances.sciencemag.org/cgi/content/full/3/6/e1602536/DC1)

- movie S1 (.wmv format). Mechanical deformation causes priming of resting neutrophils.

- movie S2 (.wmv format). Mechanically induced depolarization of PMN (fMLP-treated).
- movie S3 (.wmv format). Mechanically induced depolarization of PMN (GM-CSF–treated).
- movie S4 (.wmv format). Mechanically induced depolarization of PMN in OS without thermal effects.
- movie S5 (.avi format). RvE1-induced recircularization of mechanically polarized PMN.
- movie S6 (.wmv format). Confirmation of OS results with MMM.
- movie S7 (.wmv format). Activation of some resting cells in MMM constrictions.

Supplementary Materials and Methods

Ethics Statement

We obtained ethical approval for the study "Function and Fate of Human Granulocytes" from the ethics committee,East of England, Cambridge Central (Study No. 06/Q0108/281) and the continuation study “Zytomechanische Charakteristika humaner Immunzellen im Rahmen von Infektion und Autoinflammation” from the ethics committee of the Technical University of Dresden, Germany (Study No. EK 89032013). Informed consent of every study participant was obtained prior to blood donation.

Preparation of primary human neutrophils

Primary human neutrophils were isolated from the peripheral blood of healthy adult volunteers (male and female), using dextran sedimentation, followed by centrifugation through discontinuous plasma Percoll™ gradients. Purified neutrophils, derived from the 42%/51% Percoll™ interface, were harvested, washed and re-suspended at 5 x 10^6 cells/ml in phosphate buffered saline (PBS) with $CaCl_2$ and $MgCl_2$ (PBS++; Sigma D8662). The purity of isolated neutrophils prepared in this manner and used for the experiments was >95% as assessed by methanol fixed and REASTAIN Quick-Diff stained cytospin preparations. By Trypan Blue exclusion, purified neutrophils were > 99% viable. During the establishment of the isolation procedure, neutrophils were also assessed to be quiescent or unprimed with respect to basal and fMLP-stimulated superoxide anion release and if maintained in plasma circulate in an entirely physiological manner when re-injected in vivo.

Chemical-induction of neutrophil priming/activation

For the priming/activation of neutrophils using fMLP (Sigma-Aldrich 47729) at a concentration of 100 nM, we resuspended hPMN in PBS++ at a density of 5 x 10^6 cells/ml. The Eppendorf vial was placed in a thermomixer (Eppendorf 022670107) at 37°C and shaken for 10 minutes at 400 rpm. For the priming/activation of neutrophils using GM-CSF at 10 ng/ml (Sigma-Aldrich). Neutrophils were incubated at 37°C and shaken for 30 minutes at 400 rpm. Neutrophil priming peaks differently depending on stimuli and starts to return to normal over a period of approximately 30 – 120 minutes (*4*). While fMLP peaks quickly and strongly, and decays relatively fast, GM-CSF elicits a more delayed, but also more stable response. Over the course of the experiment, there was an increasing number of round, spontaneously de-primed cells in the sample, which we excluded from our morphological and mechanical analysis. With GM-CSF

there were more amoeboid cells at later times in comparison to fMLP. Evaluating exclusively the clearly still activated cells, the results were consistent.

Fluorescence imaging

Nuclear and cytoplasmic staining.

The nuclei of neutrophils were stained using SYTO® 61, a red fluorescent nucleic acid-binding dye, at a 5 nM final concentration, in order to observe the nuclear lobulation. The cytoplasm was stained using Mitotracker Orange (green dye for mitochondria and entire cytoplasm). Cells were placed in indented glass slides and imaged using a confocal microscope (LSM 700, Carl Zeiss, Germany).

Actin imaging using Phalloidin.

To stain actin filaments in both primed and resting neutrophils, cells (while still in suspension) were fixed with 4% formaldehyde in PBS solution for 20 minutes at room temperature. Thus, the cells were ‘‘frozen’’ in the suspended state before they had any time to start adhering to the substrate, to minimize in vitro effects prior to the quantitative fluorescence imaging and assessment of circularity. About 100 µl of the suspended fixed cells was then seeded onto a PDL (poly-D-lysine)-coated glass slide for 20 minutes at room temperature. We then incubated the cells with 0.1% Triton X-100 diluted in PBS- (PBS without $CaCl_2$ and MgCl) for 5 minutes, for permeabilization. Cells were subsequently stained with Alexa-Fluor 488® phalloidin (Life Technologies) diluted in a 1% BSA solution in PBS for a final concentration of 165 nM, and incubated for 20 minutes at room temperature in the dark. The sample was then washed three times with PBS-. Cells stained for actin were imaged using LSM 700 confocal microscope (Carl Zeiss, Germany) with a Plan Neofluar 40x 1.3 NA oil objective (Carl Zeiss) as well as a Plan-Apochromat 20x 0.8 NA air objective (Carl Zeiss) to obtain larger fields of view. Gain settings were kept constant for all samples of a given experiment and about 10 images from 10 different regions of the glass slide were analysed in each experiment.

Assessment of priming/de-priming using immunofluorescence.

CD11b (also β2 integrin, Mac-1, CD18) and CD62L (also L-selectin, LAM-1 und MEL14), and their inverse regulation, are standard molecular indicators of neutrophil priming and resting, respectively (*56*, *57*). Both resting and primed neutrophils were treated with FITC-conjugated monoclonal anti CD11b (Invitrogen) for the assessment of priming. 5 µl of the undiluted antibody

was added to 500 µl of cells at a density of $5x10^6$ cells/ml. Confocal imaging of the resting cells and the fMLP or GM-CSF primed cells were performed (LSM 700) after 15 minutes antibody staining. To ensure that gain settings, percentage laser transmission, pin-hole size, etc, were kept constant for all samples of a given experiment and for various independent experiments, the LSM Zeiss settings, were saved and reused each time. About 10 images from 10 different regions of the glass slide were analysed in each experiment.

For the assessment of de-priming, pacific blue-conjugated monoclonal anti CD62L (Invitrogen) was used. This antibody was chosen to enable simultaneous imaging of various fluorophores using three different laser wavelengths for excitation: red (639 nm) for the Syto 61 stained nuclei, green (488 nm) for FITC-conjugated CD11b and blue (405 nm) for pacific blue-conjugated CD62L). Confocal images were taken within 5-10 minutes after addition of 5 µl of undiluted CD62L antibody in 500 µl suspension of 5 $x10^6$cells/ml. We quantified and compared fluorescence intensities using the Zen 2012 Software as provided for LSM 700 confocal microscopy (Carl Zeiss).

Measurement of ROS-production in PMNs (Chemoluminescence)

Quantification of intracellular reactive oxygen species (ROS) production in resting and activated neutrophils was done via luminol-enhanced chemoluminescence. As per the manufacturer's protocol (Sigma), we first obtained an optimal luminol final concentration of 5 mM by selecting from a range of 5 mM to 50 mM trials (done in steps: 5, 10, 20, 30 40, 50 mM). We used a Single Tube Luminometer, (Promega BioSystems TD-20/20) operated at 37°C, and 300 - 650 nm range of light wavelength.

Measurement of refractive index of neutrophils

Using a digital holographic microscope (DHM) and protocols developed in our previous work (*22*, *23*), we measured the average refractive index (RI) of neutrophils to be 1.387+/-003 (for N = 1, 28 cells, RI = 1.3859 and for N = 2, 26 cells, RI = 1.3882). There was statistically no significant difference between the RI of resting neutrophils (completely round) and the neutrophils that were just beginning to be activated (polarized and slightly amoeboid cells). Obviously, the RI of neutrophils at later stages of activation when cells showed pronounced amoeboid morphology, with several pseudopodia, could not be measured accurately by our setup owing to the need to know the geometry of the cell in order to extract the RI from the phase change in the laser light induced by the cell.

Microfluidic microcirculation mimetic (MMM)

To mimic the advection of cells in capillaries, many of which have constrictions smaller than blood cell diameters, we developed the MMM. After conceptualization, the design was undertaken using AutoCAD software for three variants of the MMM: one without constrictions (constant width of 15 μm) and the others with 5 and 7 μm as the smallest constriction widths (maximum width 15 μm). All variants had a constant height of 15 μm. A polyester photomask of the design was printed commercially (Photo Data and J.D. Photo-Tools, UK). Using the photomask, the master moulds were fabricated at the Institute of Semiconductors and Microsystems of TU Dresden, following standard photolithographic techniques. For soft lithography, PDMS (Sylgard 184, Dow Corning) was degassed by centrifugation (1200 rpm for 20 minutes), poured onto moulds, and baked for about 20 minutes at 100°C. Inlet and outlet holes were punched (Harris Uni-core, 1.5 mm hole) into the cut and peeled PDMS chip. The chips were bonded to 22 mm glass cover slips (Nr 1, Marienfeld, Germany) using oxygen plasma (Plasma Cleaner PDC-32G, Harrick Plasma, USA). The inlet and outlet of the devices were connected to a computerized air pressure control system (MFCS-FLEX, Fluigent, France) using custom-cut pipette tips (Eppendorf, Germany) in which about 20 – 40 μl of cell suspension was introduced for each experiment. Cells were advected sequentially through the microchannels using driving pressures within the physiological range. For imaging, the device was mounted on an Olympus IX-70 inverted microscope (Japan) with 20x and 40x phase contrast magnification objectives for measuring cell size and 5x bright field objective for measuring the transit time through the entire device. A FireWire CCD camera (DMx 21BF04, Imaging Source, Germany) was used to record advection videos at a frame-rate of 60 frames per second. This relatively low frame rate was sufficient since transit times through the entire device were always at least an order of magnitude longer than the frame interval of 0.0167 s. Transit times were extracted from the videos using algorithms implemented in Matlab (Mathworks, Natwick, MA).

RvE1 administration to mechanically primed neutrophils in the OS

We built a new optical stretcher setup specific for this investigation, in which neutrophils are not trapped inside a glass capillary, but directly between the two opposing optical fiber ends of the optical stretcher (a so-called “open” setup). This configuration permits the controlled release and administration of drugs from a micropipette in immediate vicinity of the cell while it is trapped by the laser beams. Releasing a constant flow of 10 nM RvE1 over 5 minutes to a mechanically primed neutrophil in the trap did not show any effect. However 100 nM RvE1 resulted in a beginning re-circularization of the neutrophil within 1-2 minutes. The total process of re-

circularisation spanned up to 20 minutes of constant drug infusion. In case of earlier termination of the drug release the re-circularization effect did not complete and the neutrophils re-polarized (see movie S5).

Viability testing

The viability of cells in the MMM was tested by collecting cells from the outlet reservoir following advection and running both Trypan Blue dye exclusion tests and an apoptosis assay. In order to show, that the basic operation of MMM can be performed without harming the cells, we flowed resting neutrophils through the MMM at a high pressure of 300 mbar, collected the cells from both inlet and outlet reservoirs, stained with Trypan Blue, and counted the dead (stained) cells with a hemacytometer. Viability was only slightly reduced from 98.1/98.3% to 94.3/97.4% in two repeats (see fig. S9 left).

In order to test whether cells are still viable after chemical (fMLP) stimulation and mechanical de-polarization using the MMM, and to exclude that the phenomenon we observe is due to cell death, we stained cells after mechanical de-polarization in the MMM outlet for Annexin V/Propidium Iodide, and imaged them on a confocal microscope (LSM 700, Carl Zeiss, Germany). No staining was detectable, in contrast to the positive controls of cells that had been killed intentionally by heating to 56 ºC for 5 min followed by incubation at 37°C for 30 minutes (see fig. S9 right).

Similar viability tests after mechanical de-polarization with the OS could not be performed because it is not practically possible with the setup available to just collect the few mechanically de-polarized cells from the microfluidic channel, which also contains very large numbers of cells that have not been trapped and mechanically stimulated. However, the viability of other cells in even extended compliance measurements with the optical stretcher have been confirmed previously (see *58*, *59*).

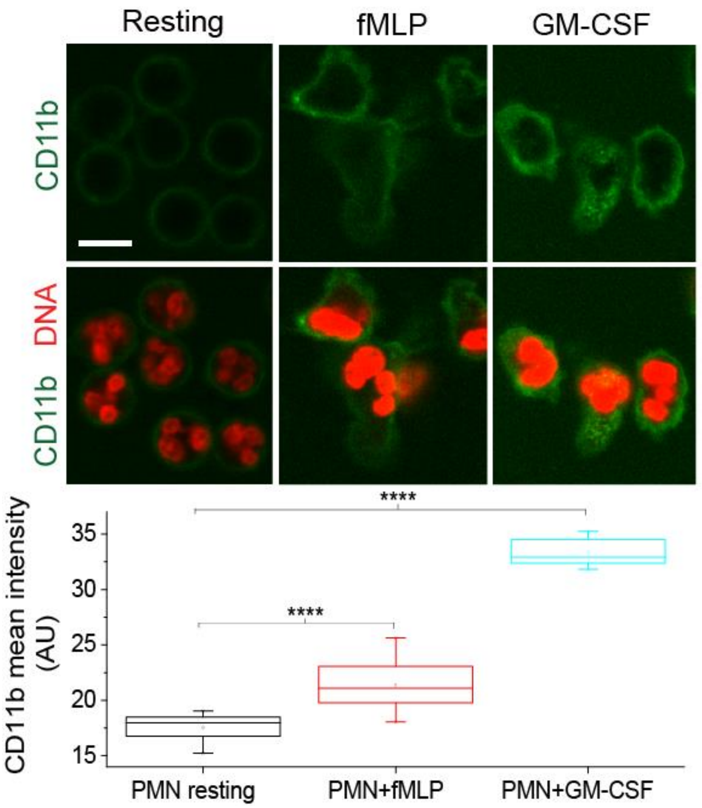

fig. S1. Chemical priming of resting neutrophils leads to increase in CD11b expression. Confocal images of FITC-conjugated CD11b+ neutrophils with Syto-61-dye-stained DNA. GM-CSF and fMLP-activated neutrophils have significantly higher CD11b expression than resting neutrophils. The **** indicate significant differences (p values < 0.0001). Scale bar is 5 µm.

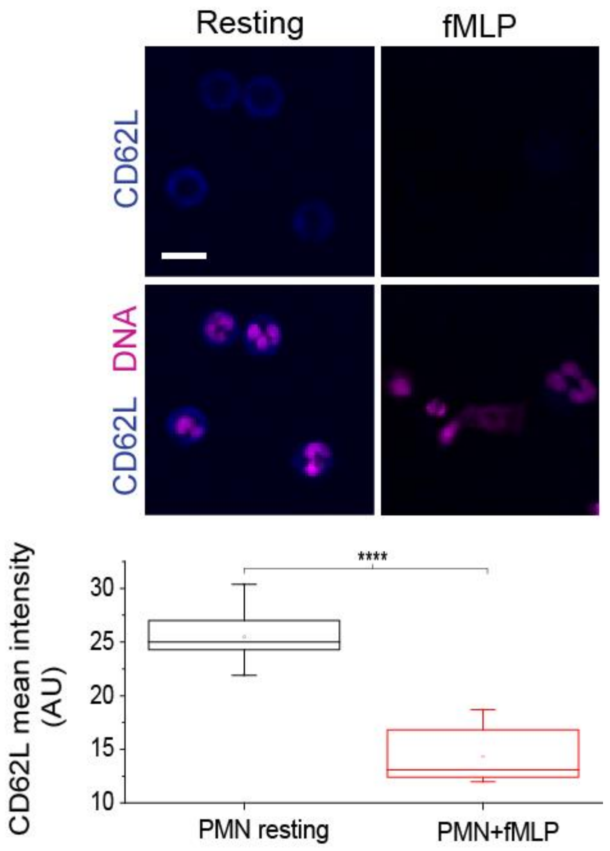

fig. S2. Chemical priming of resting neutrophils leads to decrease in CD62L expression. Confocal images of pacific blue-conjugated CD62L+ neutrophils with Syto-61-dye-stained DNA. Resting neutrophils have significantly higher CD62L expression than fMLP-primed (shown) as well as GM-CSF-primed (not shown) neutrophils. The **** indicate significant differences (*p* values < 0.0001). Scale bars are 5 µm.

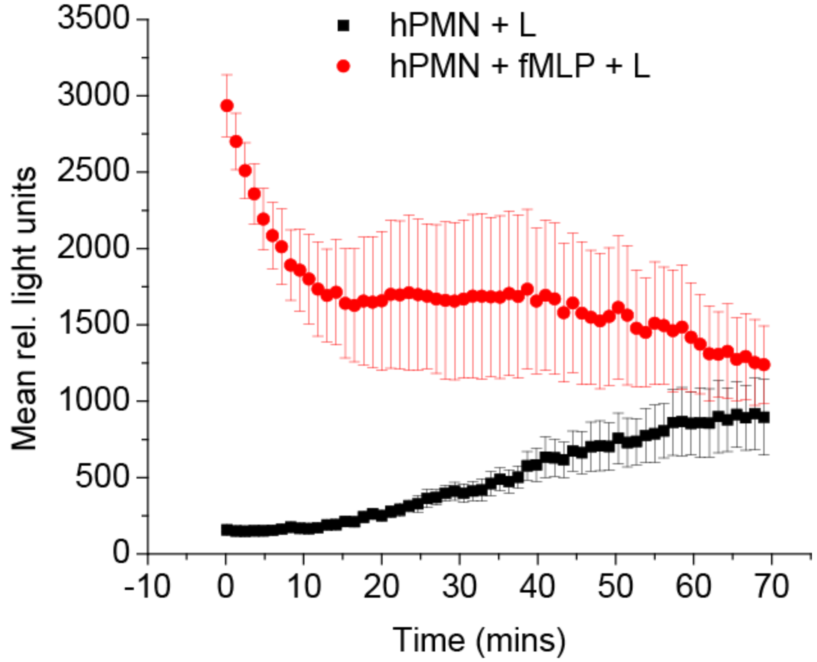

fig. S3. Higher ROS production in activated neutrophils compared to resting neutrophils. Activated neutrophils produce significantly more ROS than resting neutrophils. At bulk cell level intracellular ROS production in activated neutrophils is increased.

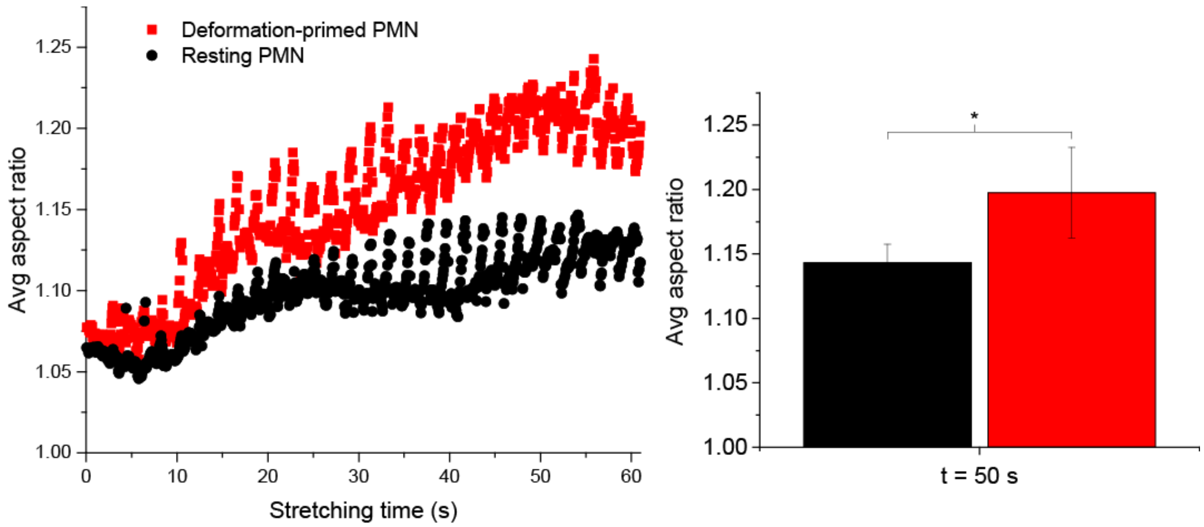

fig. S4. Average aspect ratio during mechanical priming of neutrophils. (Left) Averaged temporal evolution of aspect ratio during multiple stretching of round, resting PMNs in the OS using sinusoidal pulses of laser power (as in Fig 3*A* for the evolution of strain). Those cells that become stiffer (11 out of 25 cells) also acquire the morphology of primed/activated cells leading to increased aspect ratios over time (red) compared to those that remain round and resting (black; 14 out of 25 cells). The distinction between the two groups is made via post-analysis visual sorting based on the morphological parameters established (Figure 1). The increase in aspect ratio of the resting cells is due to their passive deformation by the optical stretching. This result is representative for $N = 3$ repeats. (Right) Bar graphs of the average aspect ratios of the two groups shown on the left at $t = 50$ s (equivalent to the values for strain in Fig. 3*B*). * indicates significant difference with $p < 0.05$.

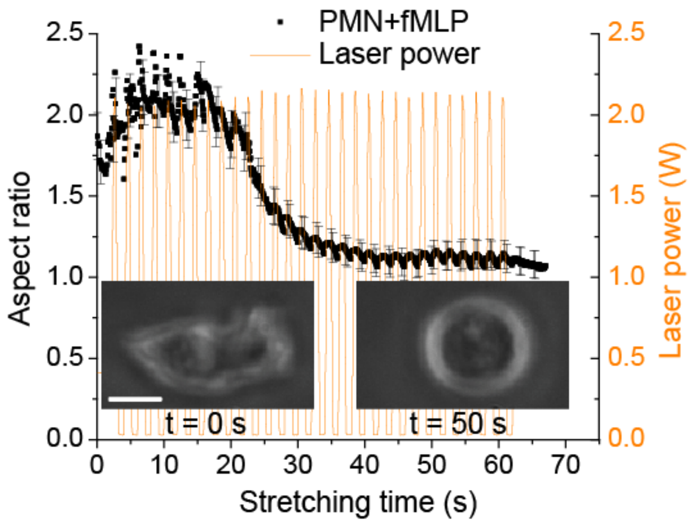

fig. S5. Mechanically induced depolarization of a chemically primed neutrophil. Repeated sinusoidal pulses of laser power (yellow line) cause a single fMLP-primed neutrophil (inset picture, t = 0 s) to de-polarize as evidenced by the retraction of pseudopods and subsequent decrease in the aspect ratio (inset picture, right), forming almost a perfectly round cell with an aspect ratio close to 1. Please note that the aspect ratio close to 2 at the beginning of the experiment constitutes an extreme case in our observations. This shows that also strongly activated neutrophils, with strong amoeboid morphology and clear pseudopod formation, can be de-polarized within 60 s.

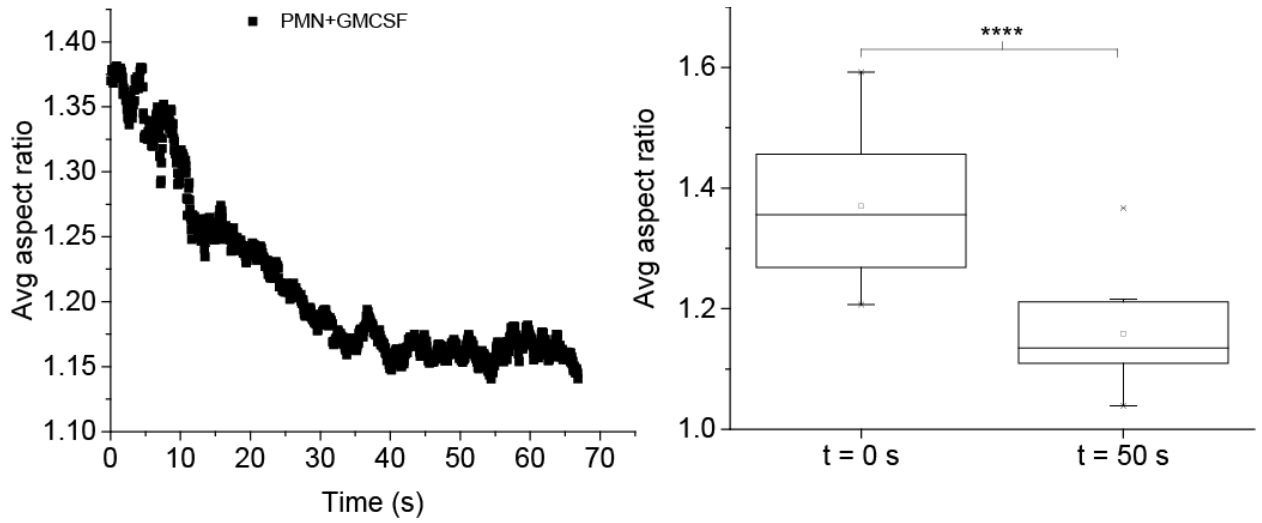

fig. S6. Mechanically induced depolarization of GM-CSF–primed neutrophils. Repeated sinusoidal pulses of laser power cause GM-CSF-primed neutrophil to de-polarize as evidenced by the retraction of pseudopods and subsequent decrease in the average aspect ratio, forming round cells, as shown in Fig. 3C with fMLP-primed neutrophils. (Left) Averaged temporal evolution of aspect ratio under sinusoidal stretching of $n = 13$ cells. Here, this occurs for about 60% of the primed cells. This result is representative for $N = 3$ repeats. (Right) Box plots of the average aspect ratios of the cells shown on the left at $t = 0$ s and $t = 50$ s. **** indicates significant difference with $p < 0.0001$.

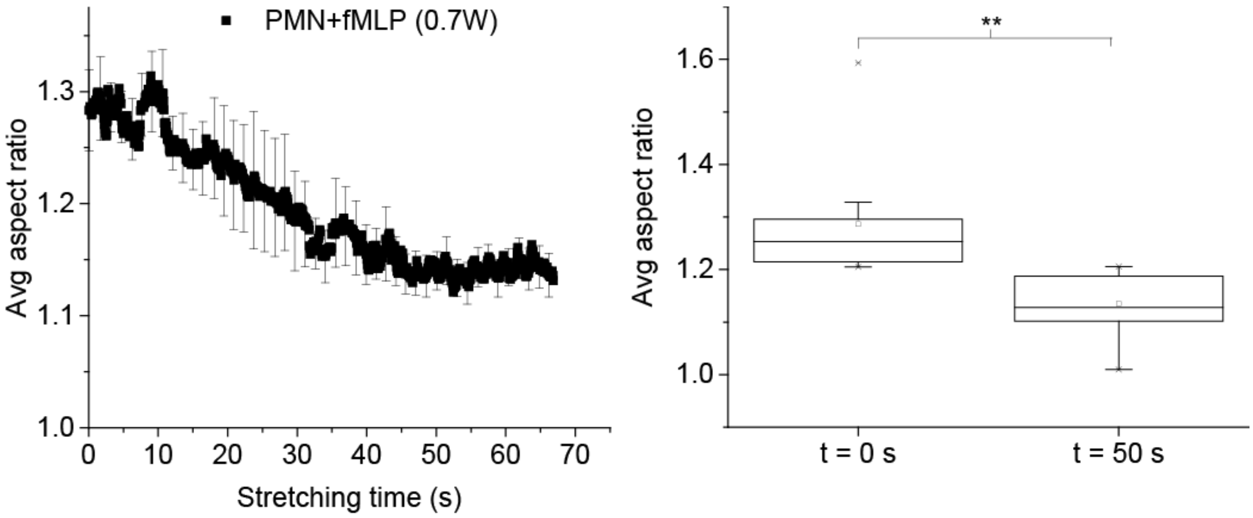

fig. S7. Mechanically induced depolarization of a chemically primed neutrophil at a lower laser power. (Left) Averaged temporal evolution of aspect ratio (*n* = 10 cells) of fMLP-primed neutrophils under sinusoidal stretching with 25% lower stress magnitude (0.7 W/fiber, instead of 0.9 W/fiber as in all other experiments). The de-polarization seems to be delayed compared to the 0.9 W/fiber case. (Right) Box plots of the average aspect ratios of the cells shown on the left at $t = 0$ s and $t = 50$ s. ** indicates significant difference with $p < 0.01$.

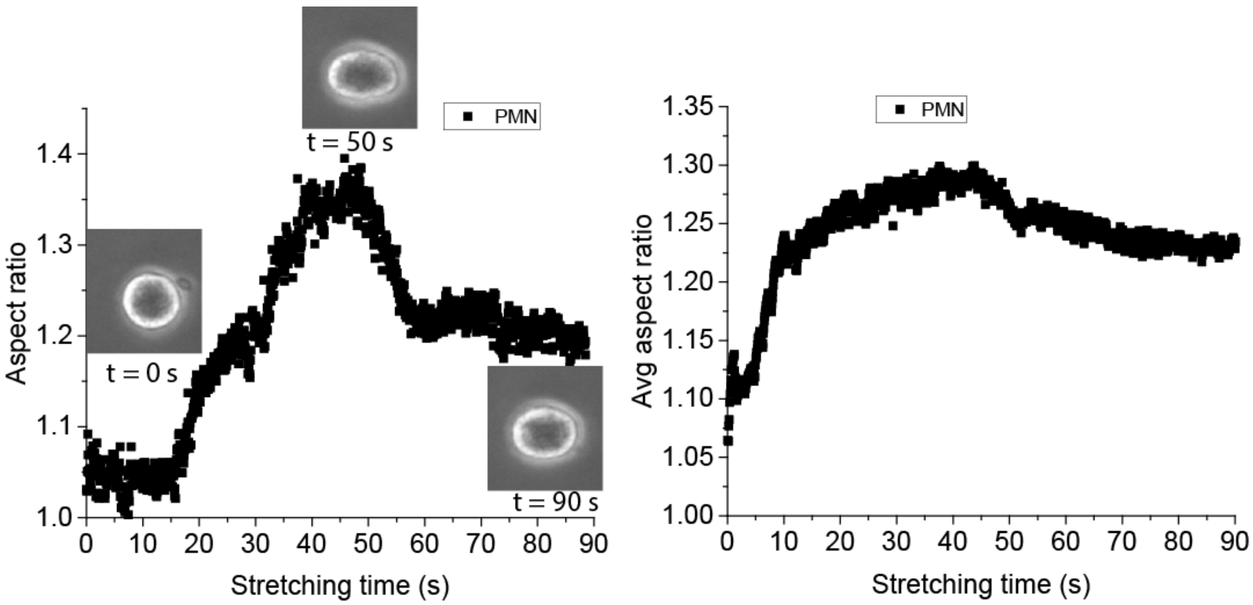

fig. S8. Mechanically induced priming and depolarization of resting neutrophils. (Left) Repeated sinusoidal pulses of laser power cause a single resting neutrophil to become primed by 40 – 50 s, as evidenced by the increase in aspect ratio, and then to de-polarize within the following 15 s. Please note that the residual higher aspect ratio at the end of the experiment, compared to Fig. 3*C* and *D*, S5 to S7, might be caused by the passive, viscoelastic deformation occurring over a much longer period of time. In three repeats, we found that 4 out of 28 (14%), 3 out of 31 (10%) and 12 out of 19 (63%) resting cells showed both mechanical priming and mechanical depolarization in one experiment. In all cases in which we tried to generate another mechanical priming event for up to 400 s we observed none. (Right) Time evolution of average aspect ratio of mechanically primed and de-polarized cells (n = 17) cells, which is representative of N = 3 experiments.

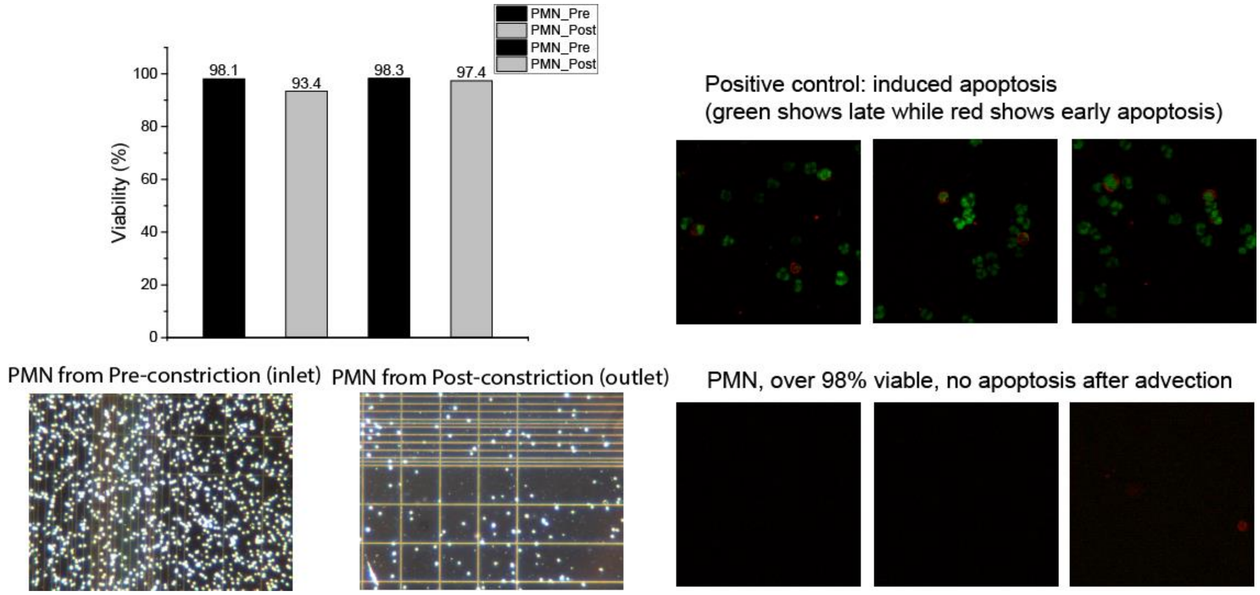

fig. S9. Viability tests using trypan blue and annexin V/propidium iodide. (Left) (Left) Cells are viable during a normal operation of the MMM tested by flowing resting neutrophils through the MMM at a pressure of 300 mbar (high pressure compared to the de-priming pressure of 50 mbar), collecting cells from the outlet reservoir, staining with Trypan Blue and counting of the dead (stained) cells using a hemocytometer (lower left). Viability was only slightly reduced from 98.1/98.3% to 93.4/97.4% and the reduction was not statistically significant ($p > 0.05$). (Right) The viability of fMLP-primed and mechanically de-polarized neutrophils was tested by staining cells after mechanical de-polarization in the MMM outlet for Annexin V (green) and PI (red). The top panels shows thermally induced cell apoptosis as a control while in the bottom panels PMN viability following advection in MMM was about 98 ± 2 %.

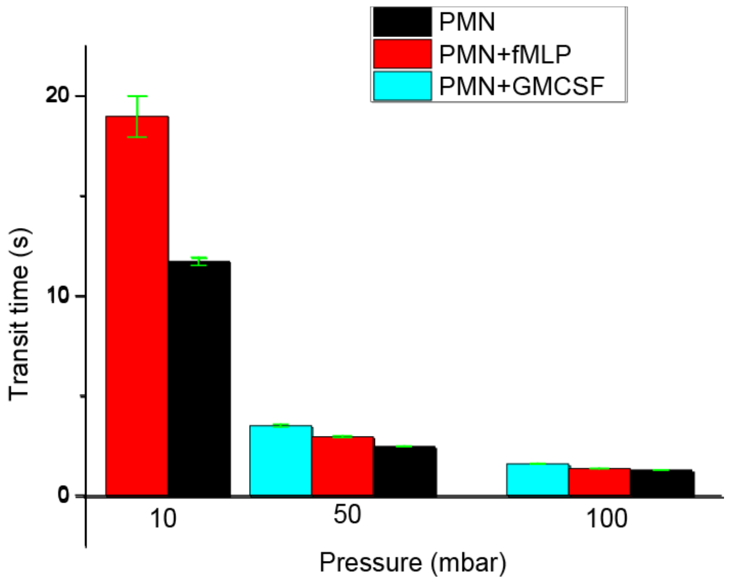

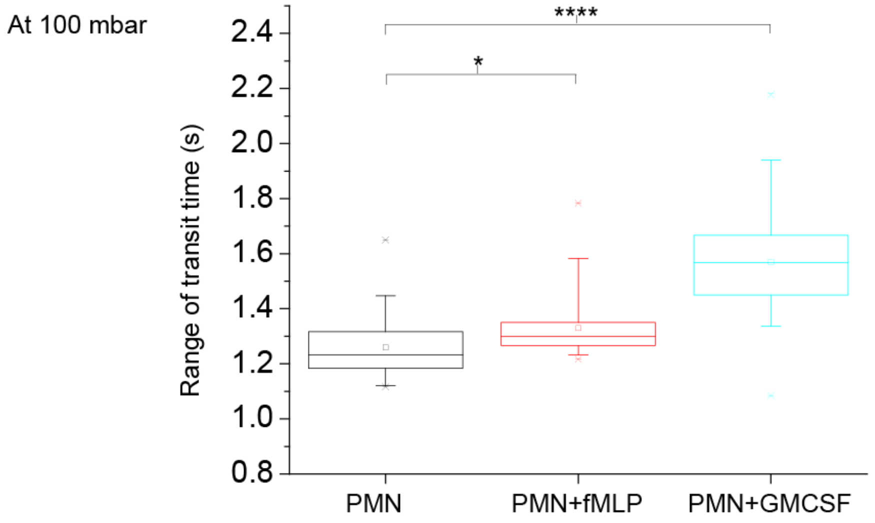

fig. S10. Delayed transit of activated cells in MMM at a lower temperature of 24°C. Bar graphs of average transit times of resting PMN, fMLP-treated and GM-CSF treated cells show that the primed cells have significantly longer transit times than resting PMN, at 24°C, at 10, 50 and 100 mbar. The box plot shows the 100 mbar result (n = 63, 68 and 103, respectively, for non-, fMLP- and GM-CSF-treated cells. **** and * indicate significant differences with $p < 0.0001$ and < 0.05, respectively.

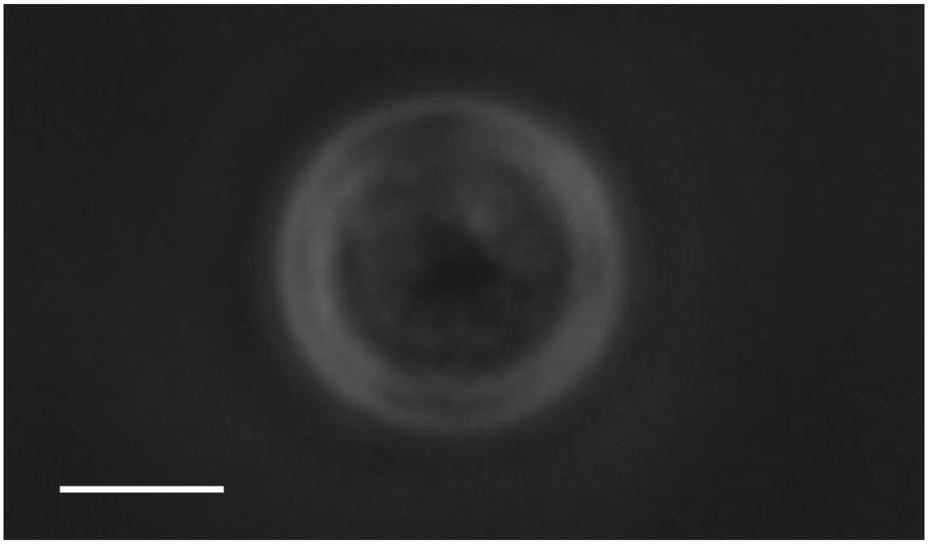

movie S1. Mechanical deformation causes priming of resting neutrophils. The video is presented in real time. Scale bar is 5 µm. Repeated stretching of the resting cells in the OS gradually leads to a change of shapes from round to the amoeboid phenotype in about 40% of the cells within 60 s of 0.5-1 s pulsatile/sinusoidal stretching.

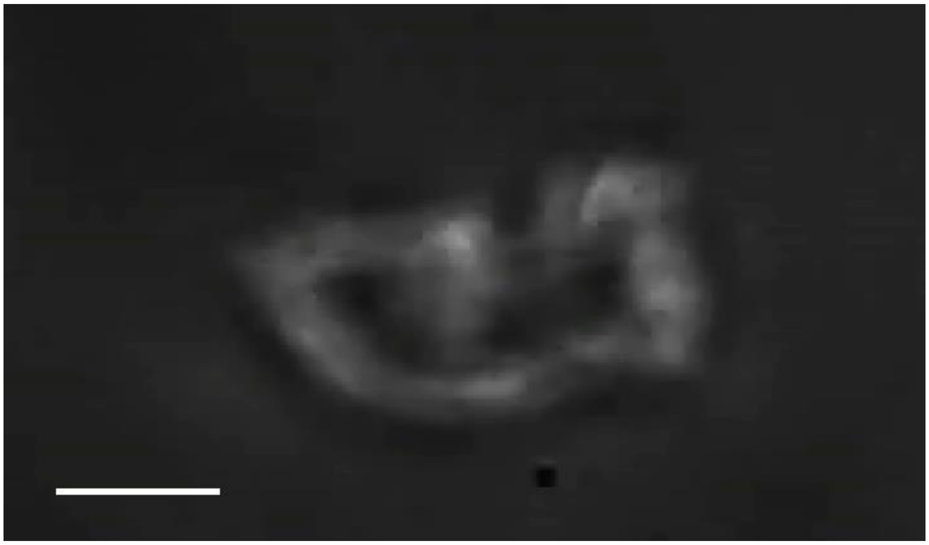

movie S2. Mechanically induced depolarization of PMN (fMLP-treated). The video is presented in real time. The fMLP-primed cells show decrease in aspect ratio towards that for resting cells, as they are repeatedly stretched in the OS, using 0.5-1 s pulsatile/sinusoidal stretching. Scale bar is 5 µm.

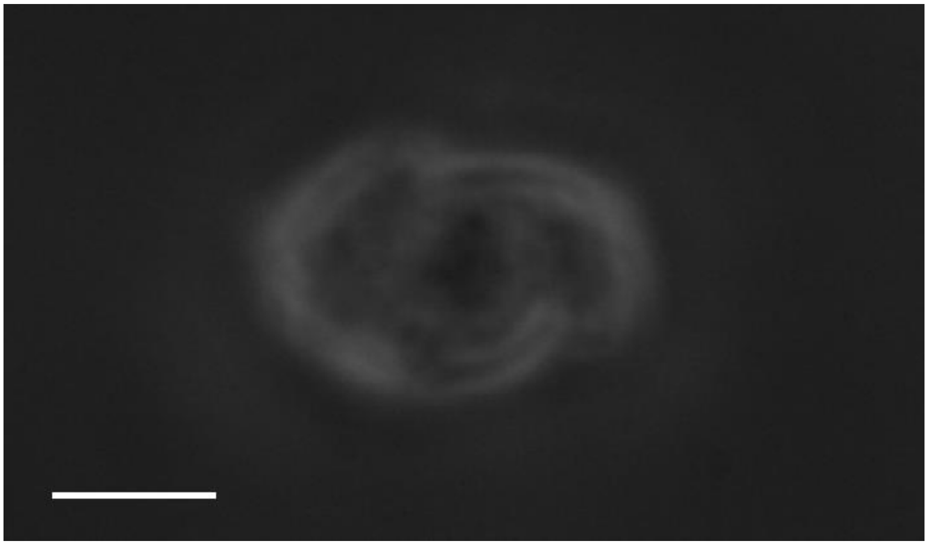

movie S3. Mechanically induced depolarization of PMN (GM-CSF–treated). The GM-CSF-primed cells also round up and gradually take the morphology of resting cells, as they are repeatedly stretched in the OS, using 0.5-1 s pulsatile/sinusoidal stretching. Scale bar is 5 μm. The video is presented in real time.

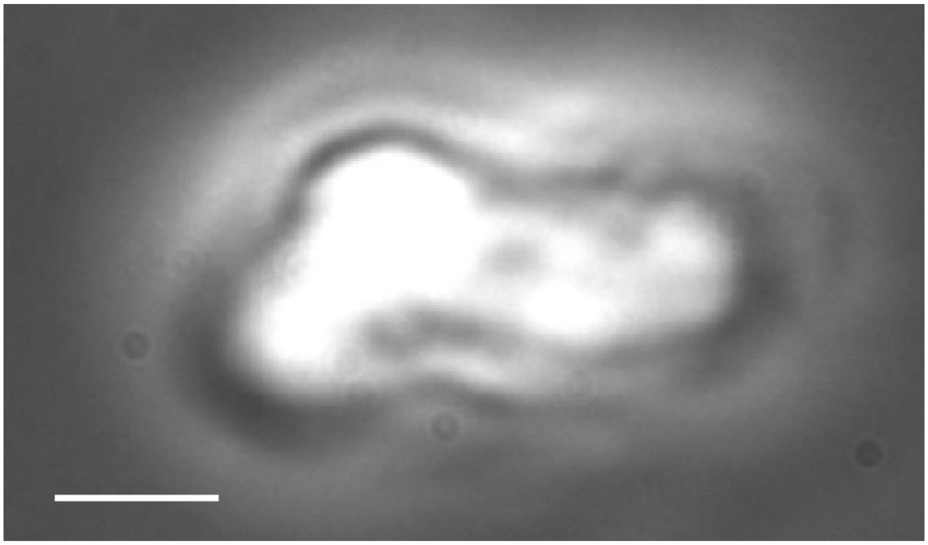

movie S4. Mechanically induced depolarization of PMN in OS without thermal effects. Using a 780 nm OS in which the temperature increase is only a few°C as compared to the 12°C/W with a 1064 nm OS, primed cells also round up during pulsatile/sinusoidal stretching. Scale bar is 5 µm. The video is presented in real time.

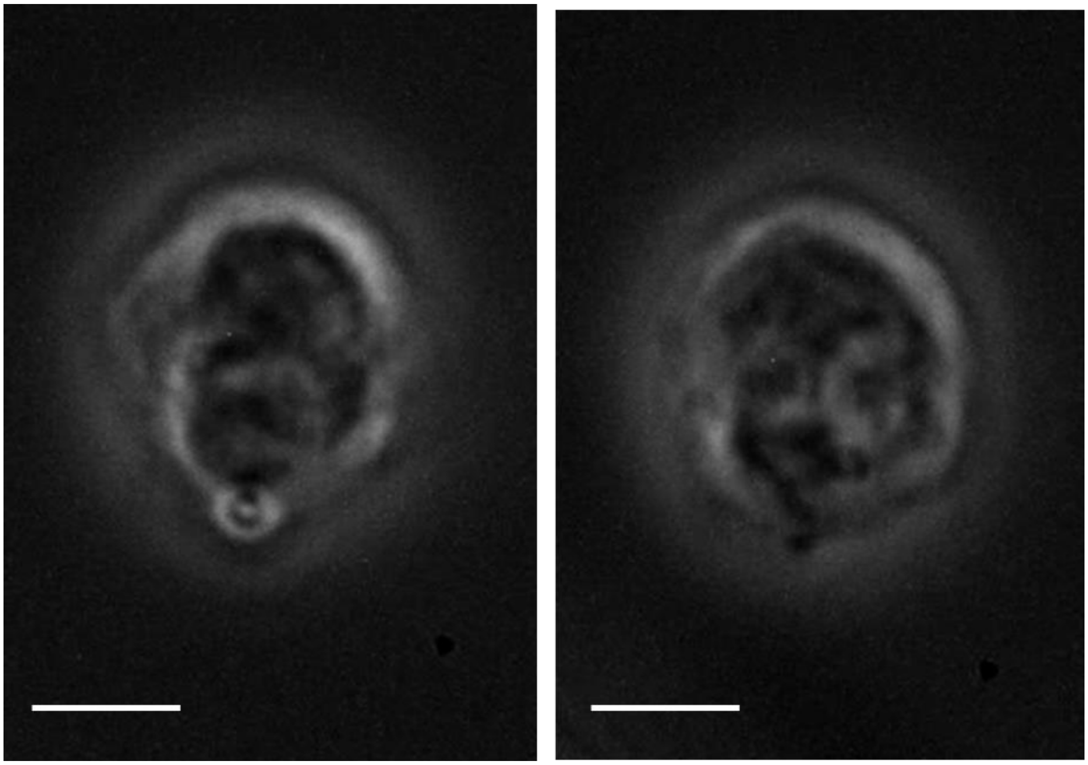

movie S5. RvE1-induced recircularization of mechanically polarized PMN. The neutrophil is trapped by two counter-propagating laser beams directly between the two opposing optical fiber ends in a so-called “open” OS setup. RvE1 is released from a micropipette in immediate vicinity to the neutrophil. During a constant flow of 100nM RvE1 the mechanically polarized neutrophil (left) re-circularized within 20 min (right). Scale bar is 5 µm.

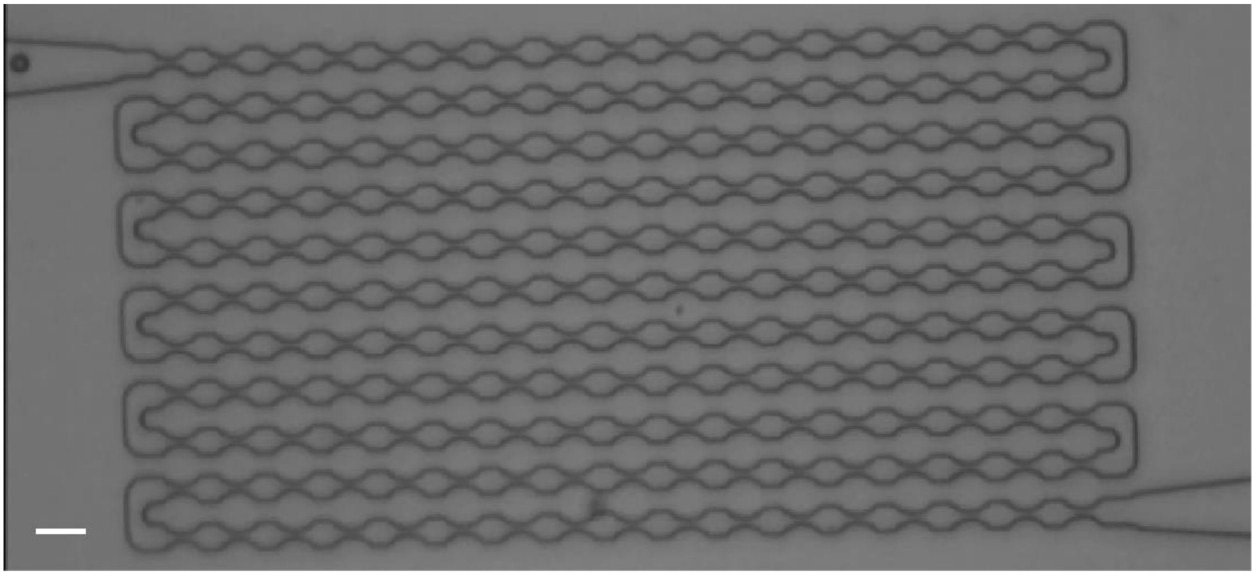

movie S6. Confirmation of OS results with MMM. The video is presented in real time. A constant pressure difference, maintained between the inlet and the outlet, was used to advect cells through the device, one cell at a time, for measurement of transit time. Scale bar is 20 µm.

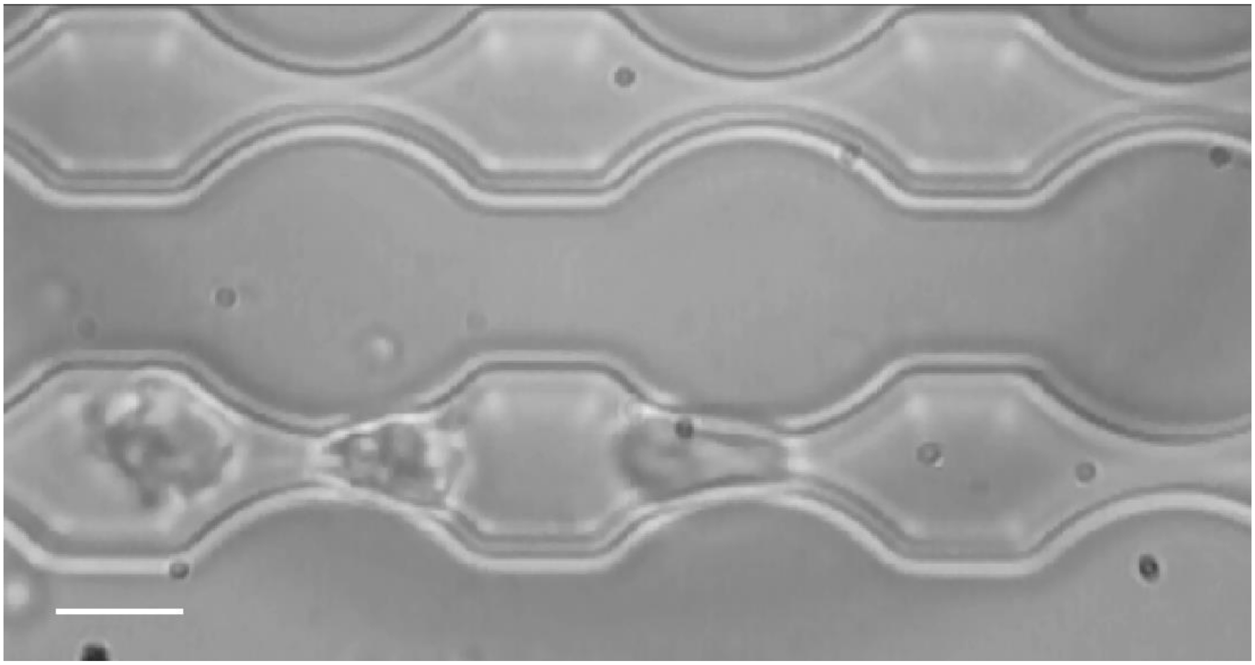

movie S7. Activation of some resting cells in MMM constrictions. At very low pressures (1 – 5 mbar), advection of resting cells through MMM back and forth, led to activation of some cells. Scale bar is 10 μm.

Other materials S1 to S3. Consent forms and questionnaire for blood donors.

Other S1. Consent Form (Cambridge, England)

Consent Form, *09/11/2012, Version 4*
Principle Investigator: Professor Edwin Chilvers

UNIVERSITY OF CAMBRIDGE
Department of Medicine
School of Clinical Medicine

CONSENT FORM

Patient Identification Number for this Trial:

TITLE OF PROJECT: Function and Fate of Human Granulocytes

PRINCIPAL INVESTIGATOR: Professor Edwin Chilvers

Name of Researcher: ....

Please initial box

1. I confirm that I have read and understand the information sheet dated *09/11/2012 (version 4)* for the above study. I have had the opportunity to consider the information, ask questions and have had these answered satisfactorily. ☐

2. I understand that my participation is voluntary and that I am free to withdraw at any time, without giving any reason, and without my medical care or legal rights being affected. ☐

3. I agree to a blood sample for use in this study. ☐

4. I understand that sections of any of my medical notes may be looked at by doctors involved in this study and from regulatory authorities where it is relevant to my taking part in research. I give permission for these individuals to have access to my records. ☐

5. I agree to take part in the above study. ☐

6. ***I confirm that I have not taken part in another study during the last 3 months*** ☐

Name of Patient	Date	Signature
Name of person taking consent (if other than researcher)	Date	Signature
Researcher	Date	Signature

When completed, 1 for participant; 1 for researcher site file; 1(original) to be kept in study file

Other S2. Consent Form (Dresden, Germany), original

Universitätsklinikum Carl Gustav Carus
Klinik und Poliklinik für Kinder- und Jugendmedizin

Universitätsklinikum
Carl Gustav Carus Dresden
an der Technischen
Universität Dresden

STUDIE:
„Zytomechanische Charakteristika humaner Immunzellen im Rahmen von Sepsis und Autoinflammation"

Patientenaufkleber

EINVERSTÄNDNISERKLÄRUNG

Ich bestätige, dass ich die Studienaufklärung zu der oben genannten Studie gelesen und verstanden habe. Ich habe die Möglichkeit gehabt, die Informationen zu überdenken und Fragen zu dieser Studie zu stellen, die zu meiner Zufriedenheit beantwortet wurden.

Ich verstehe, dass meine Teilnahme an dieser Studie auf freiwilliger Basis erfolgt und dass ich jederzeit ohne Angabe von Gründen meine Teilnahme an der Studie widerrufen kann. Eine Nichtteilnahme oder ein Widerruf meiner Teilnahme an der Studie hat keinen Einfluss auf die medizinische Behandlung.

Ich verstehe und stimme zu, dass die Ergebnisse der Analysen, die während der Studie gewonnen werden, von den an der Studie teilnehmenden Wissenschaftlern ausgetauscht und besprochen werden.

Ich verstehe, dass meine Blutprobe für die Untersuchungen im Rahmen der Studien verwendet wird.

Ich stimme einer Teilnahme an der Studie zu.

______________________	______________	________________________
Name	Datum	Unterschrift
______________________	______________	________________________
Name des/der aufklärenden Arztes/Ärztin	Datum	Unterschrift

Original im Studienordner, 1 Kopie für Studienperson

Other S3. Questionnaire for blood donors prior to donation used in Dresden, Germany (translated to English)

STUDY

"Cell mechanical characteristics of human immune cells in infection and inflammation"

Questionnaire for healthy blood donors

Completed by the study doctor:

Study number: |__||__| |__|__| |__|__|

Date of blood donation: |__||__| |__|__| |__|__|

Age of the study participant: |__| |__| years

Gender of the study participant: O male O female

Please answer the following questions, by ticking the correct answers or underlining the appropriate statement. If you have any questions please feel free to ask the study doctor at any time.

Question 1: Do you feel healthy: O no O yes

- -

Question 2: During the last four weeks, did you have:

2.1 Infection (e. g. cold, vomiting/diarrhoea, urinary tract infection): O no O yes

2.2 Dental or surgical intervention: O no O yes

2.3 Vaccination: O no O yes; if yes, which: ______________________________

- -

Question 3: Did you receive any medical treatment during the last two month?

O no O yes, where and why? ____________________________________
____

- -

Question 4: During the last year, did you receive:

4.1 Any blood transfusion? O no O yes

4.2 Drugs of human or animal origin (e.g. blood coagulation factors, immunoglobulins, antibodies against tetanus etc.)? O no O yes

- -

Please also answer the questions at the back of this page!

<u>Question 5:</u> Do you have or ever had one or more of the following diseases?
(Please underline the appropriate disease conditions)

5.1 Cardiovascular diseases or problems with your blood circulation (z. B. blood hypertension, thrombosis, embolism, stroke) O no
O yes

5.2 Allergies, autoimmune disorders (e. g. rheumatic fever), epilepsy, diabetes, cancer?
O no O yes

5.3 Infection disease, e.g.Hepatitis A, Hepatitis B, Hepatitis C; HIV-1/2, AIDS or HTL-Virus (HTLV-1/2); tuberculosis, osteomyelitis, toxoplasmosis, measles, salmonellosis, typhoid or paratyphoid, Q-fever, Chagas-disease, brucellosis, babesiosis, leishmaniasis, leprosy, melioidosis, mumps, recurrent fever, deer fly fever (tularemia), spotted fever, Rickettsia, rubella, varicella, scarlet fever?
O no O yes

5.4 Other immune-suppressive disease, auto-immune disease or other infection disease?
which?: ____

5.3 Any other disease conditions affecting your skin, blood, brain, lung (e.g. asthma), liver, kidneys, stomach, gut, nervous system, or lymphatic system?
O no O yes

- -

<u>Question 6:</u> Did you take drugs like anti-baby-pill, painkillers (e.g. Aspirin/ASS, Ibuprofen) etc. during the last four weeks? And do you take any other drugs (e.g. drugs against hypertension, hay fever, immunosuppressive drugs like cortisone etc.)?
O no O yes

If yes, which drugs and how often do you take them?

____

____

____

.

Thank you for your study participation!
When completed, please return the questionnaire to the study doctor
